# Influence of COVID-19 quarantine on the health of adults with mild cognitive impairment

**DOI:** 10.1590/1980-5764-DN-2021-0013

**Published:** 2022

**Authors:** Laura Natalia Calceto Garavito, Jasmín Bonilla Santos, Alfredis González Hernández, Dorian Yisela Cala-Martínez, Duván Fernando Gómez Morales

**Affiliations:** 1 Universidad Cooperativa de Colombia. Neiva, Huila, Colombia.; 2 Universidad Surcolombiana. Neiva, Huila, Colombia.

**Keywords:** COVID-19, Quarantine, Cognitive Dysfunction, Personal Satisfaction, Depression, Affect, COVID-19, Quarentena, Disfunção Cognitiva, Satisfação Pessoal, Depressão, Afeto

## Abstract

COVID-19 is an infectious disease declared by the World Health Organization as a public health emergency of international concern.

**Objective:**

The objective of this article was to determine the physical, psychological, and social health conditions and mood of COVID-19 quarantine in adults with mild cognitive impairment.

**Methods:**

The sample consisted of 129 participants, most of them were healthy, but some have mild cognitive impairment. The data were collected with a questionnaire and the Yesavage Geriatric Depression Scale applied through phone calls from April to June 2020.

**Results:**

Statistically significant differences were found in the changes in sleep habits of the healthy participants (p=0.018). Intragroup comparisons of the Yesavage Geriatric Depression Scale in healthy participants were significant (p=0.010) and at the intergroup level before and after quarantine showed significant differences in pretest scores (p=0.003).

**Conclusions:**

Social isolation had a negative psychological effect on sleep habits, depressed mood, and physical health, mainly in healthy participants.

## RESUMO.

COVID-19 é uma doença infecciosa declarada pela Organização Mundial da Saúde como emergência de saúde pública de interesse internacional.

**Objetivo:**

O objetivo deste artigo é determinar as condições de saúde física, psicológica, social e de humor na quarentena de COVID-19 em adultos com comprometimento cognitivo leve.

**Métodos:**

A amostra foi composta de 129 participantes, a maioria saudável, mas alguns com comprometimento cognitivo leve. Os dados foram coletados por meio de questionário e da escala de depressão geriátrica de Yesavage, aplicada por meio de ligações telefônicas no período de abril a junho de 2020.

**Resultados:**

Diferenças estatisticamente significantes foram encontradas nas mudanças nos hábitos de sono dos participantes saudáveis (p=0,018). As comparações intragrupo da escala de depressão geriátrica de Yesavage em participantes saudáveis foram significativas (p=0,010) e, no nível intergrupo, antes e depois da quarentena mostraram diferenças significativas nos escores do pré-teste (p=0,003).

**Conclusões:**

O isolamento social teve efeito psicológico negativo sobre os hábitos de sono, humor deprimido e saúde física, principalmente em participantes saudáveis.

## INTRODUCTION

On March 11, 2020, the World Health Organization (WHO) decreed the global pandemic of the *coronavirus disease in 2019* (COVID-19)^1^, which causes physical and psychological disturbances and has a high mortality rate. On November 27, 2020, the data showed 61,079,040 cases and 1,433,516 confirmed deaths in the world; out of these, 3,277,248 cases and 45,149 deaths belong to the American continent^2^. According to the Ministry of Health, Colombia reported 49% of the deaths^3^.

During the global health crisis, physical health consequences and medical complications have received more attention than the pandemic effects on mental health of adults above 60 years^4^; those who have comorbidities with chronic diseases have a higher risk of experiencing mental, cognitive, behavioral, emotional, (e.g., stress, anxiety, or depression) and social deterioration, as well as disturbances in sleep habits^5,6^, therby having negative consequences in everyday functioning. WHO^7^ has recommended to set social restrictions and self-care measures to guarantee the physical and mental health of the older adult population. However, these restrictions limit contact with family and friends and daily activities such as walking, talking, or attending volunteer work in groups, which have negative consequences on psychological Personal satisfaction and physical health conditions^8–13^.

This study aimed to evaluate the physical, psychological, and social Personal satisfaction conditions in adults with mild cognitive impairment (MCI) in a South Colombian study group, in the quarantine for the COVID-19 pandemic.

## METHODS

### Study design and description

This transversal descriptive study included 129 adults (54 healthy and 75 with MCI participants in the “Longitudinal Measurement of the integration function in short-term memory in patients with Mild Cognitive Impairment” project). Three measurement sessions were scheduled during of 3 years, but with the temporary suspension of the activities due to the restrictions due to COVID-19, the physical, social, and psychological well-being of the participants was assessed. For the data collection, clinical psychologists contacted the participants of the study by phone, from April to June of 2020. For the eligibility criteria, the following points were considered: (1) age ≥50 years, (2) having a landline or mobile phone, (3) being linked to the project (with evaluation from the first moment, score <9 in the Functional Evaluation Questionnaire [FAQ] or as a loss <20% in the Instrumental Skills of Daily Living [IADL]), and (4) informed consent.

### Description of the groups participating in the project

In the project, there are two groups: healthy group and group with MCI. The classification for the first group was determined under the following criteria as established by Petersen and Winblad et al.^1^: cognitive changes reported by the affected individual and/or by someone close to them^2^; Mini-Mental State Examination (MMSE) 24 and 26 and/or Addenbrooke’s Cognitive Examination (ACE) (M-1SD): age <75: 79 (cut-off), age ≥75: 60 ()^3^; memory disability^4^; independence in functional activities^5^; and lack of dementia according to the criteria for NINCDS-ADRDA (National Institute of Neurological and Communicative Disorders and Stroke and the Alzheimer’s Disease and Related Disorders Association). The inclusion criteria for healthy participants include individuals with evidence of no cognitive, functional, and neurological impairment^1^; absence of cognitive and memory disorders (MMSE>26)^2^; and living independently without difficulty (normal score on the instrumental activities of daily living [IADL] scale and in Subjective Memory Scale).

Exclusion criteria for both groups include the following points^1^: active psychiatric disorders, history of alcohol/drugs (Yesavage Geriatric Depression Scale >5, Yesavage et al.)^2^; cerebrovascular disease (Hachinski Ischemia Scale Score >4, Hachinski et al.)^3^; significant underlying medical and/or neurological conditions^4^; visual impairment^5^; and MMSE<24 (if ACE is available, MMSE is taken from ACE).

### Instruments

The questionnaire “Conditions of Personal satisfaction during COVID-19 pandemic quarantine: ages 50 and up” was created using Google Forms, and it took 20 min to answer. The questionnaire was made to identify the current situation in adults in three dimensions: physical Personal satisfaction (17 items), psychological Personal satisfaction (27 items), and social Personal satisfaction (8 items). The content validity was performed by consensus among experts (five researchers from the areas of psychology, neuropsychology, neuroscience, and epidemiology). The level of concordance of the expert’s evaluations was analyzed with the Kappa statistical index. A coefficient of 0.60 was obtained (p=0.000). This interpreted as adequate agreement by the evaluators according to the *Kappa* classification^14^.

Geriatric Depression Scale was developed by Yesavage in 1982. The purpose of this instrument is to identify depressive symptoms in adults above 60 years of age in the past 7 days. It has 15 dichotomous items (Yes/No) and a duration of 5–10 min for its application. This study used the scale to assess adults below 60 years of age because this scale has been shown to have adequate precision in identifying symptoms of depression in young adults aged 18–59 years. It has sensitivity of 72% and specificity of 97% in young adults, and a sensitivity of 86% and specificity of 91% in older adults^15^. The 15-item version has been shown to have better diagnostic accuracy and clinical utility than the 30-item version^16^.

### Statistical analysis

Data were collected and tabulated in a Google spreadsheet file and subsequently analyzed using the *Statistical Package for the Social Sciences* (SPSS) version 23. First, a description of the characteristics of the sample is presented in absolute frequencies and percentages for the categorical variables. The comparison of nominal categorical variables in the two groups was done using the chi-square statistical test, and the normality of the numerical data was evaluated using the Kolmogorov-Smirnov test, which showed nonparametric behavior. Therefore, for the intragroup comparison of the Yesavage test in the healthy group and with MCI, the nonparametric Wilcoxon signed-rank test was used. In the intergroup comparison before and after the pandemic, the nonparametric Mann-Whitney U statistic was used.

### Ethical aspects

The research complied with the ethical standards at the international and national levels for the study with human beings, such as the Declaration of Helsinki, the International Ethical Guidelines for Biomedical Experimentation in Human Beings, the Standards of Good Clinical Practices, Resolution No. 008430 of 1993 of the Ministry of Health and Social Protection of Colombia, as well as Law 1090 of 2006 Code of Ethics and Bioethics of the Psychologist. It has the endorsement of the Ethics, Bioethics, and Research Committee of the Hernando Moncaleano Perdomo University Hospital.

## RESULTS

A total of 129 people were surveyed, the mean age was 66.4 (SD=8.1), 78% were identified as female, 28% of the participants were below 60 years old, and 72% were above 61 years old. According to a previous neuropsychological evaluation, 41.9% were considered healthy and, therefore, were part of the “healthy group” and 58.1% showed symptoms of MCI. The mean age of the healthy group was 63.6 (SD=7.2) and that of the “MCI group” was 68.4 (SD=8.2). All the results obtained from the survey show the physical, psychological, and social state perceived by the participants of the two groups in the quarantine. Table 1 shows a summary of the survey responses, including a comparison between the groups (healthy and with MCI).

**Table 1:** Comparisons of chi-square test of cognitive domains between the healthy group and the group with mild cognitive impairment.

		Healthy participants (n=54)	Participants with MCI (n=75)	Statistical significance (p-value)
**Sociodemographic characteristics, n (%)**				
Age				
	51–60	23 (43)	13 (17)	
	61–70	22 (41)	32 (43)	
	71–80	8 (15)	24 (32)	
	>80	1 (2)	6 (8)	
Gender				
	Female	44 (81)	57 (76)	0.521
Has access to a pc or laptop				
	Yes	25 (51)	24 (49)	0.141
Has Internet access				
	Yes	31 (57.4)	39 (52)	0.593
	Own financial resources	32 (59.3)	49 (65.3)	0.580
	Financial difficulties due to mandatory quarantine measures	28 (51.9)	34 (45.3)	0.481
**Physical health, n (%)**				
	Has any physical illness	20 (37)	36 (48)	0.280
	Follows a specific diet	24 (44.4)	33 (44)	1.00
	Exercises	34 (63)	45 (60)	0.855
	Has items to prevent falls at home	21 (38.9)	23 (30.7)	0.352
	Received medical treatment during quarantine	9 (17.3)	22 (29.3)	0.144
	Considers illness symptoms have increased during quarantine	11 (20.4)	9 (2)	0.043
**Psychological Personal satisfaction, n (%)**				
	Emotional distress has affected daily functioning	13 (24.1)	13 (17.3)	0.379
	Reports changes in sleep habits	29 (53.7)	24 (32)	0.018
	Reports changes in memory during quarantine	13 (24.1)	20 (26.7)	0.839
**Social life, n (%)**				
	Lives with a relative, friend, or acquittance	46 (85.2)	62 (82.7)	0.081
	Shows interest for the COVID-19 virus and how to prevent it or treat it	32 (59.3)	52 (69.3)	0.265

MCI: mild cognitive impairment.

As for the physical health state, 37% of healthy participants and 48% with MCI reported chronic health conditions prior to COVID-19. When asked about individual perception regarding the physical health state, 22.5% of the participants in the MCI group and 22% of healthy members stated that they did not feel completely well. Regarding the time spent per week doing physical activity, 37% of the healthy group did not do physical activity, 27.8% dedicated less than an hour, and 35.2% did exercise for more than an hour. Meanwhile, 40% of participants with MCI did not perform physical activity, 37.3% dedicated less than an hour, and 22% did exercise for more than an hour. Among healthy participants, 5.6% stated that they had consumed alcohol and 5.6% were smokers, and among the participants with MCI, 9.3% consumed alcohol and 4% were smokers.

Regarding the psychological Personal satisfaction, the healthy participants stated that they experienced emotional changes during quarantine: 59.3% never felt discouraged, 29.6% sometimes bored, 25.9 always relaxed, 25.9 always optimistic, 24.1% nervous sometimes, and 9.3% always irritated. In the group of participants with MCI, 26.7% reported that they felt nervous at times, 4.7% almost always irritated, 41.3% sometimes bored, 54.7% never felt discouraged, 32% always relaxed, and 28% always optimistic. Statistically significant differences were found in changes in sleep habits of healthy participants (p=0.018).

In the evaluation with the Yesavage Geriatric Depression Scale, statistically significant differences were found in the intragroup comparisons in the healthy participants (p=0.010) using the Wilcoxon rank-sum test (Table 2). In contrast, when making an intergroup comparison of the Yesavage scale before and after quarantine in healthy participants and those with MCI, statistically significant differences were obtained in the pretest scores (p=0.003) using the Mann-Whitney U test (Table 3).

**Table 2:** Wilcoxon rank-sum test for intragroup comparisons.

Group	n	Yesavage scale	Median	Percentile 25	Percentile 75	p-value
Healthy	53	Pre	1.00	0.00	2.00	0.010
	53	Post	2.00	1.00	3.00	
MCI	69	Pre	2.00	1.00	3.00	0.987
	69	Post	2.00	1.00	3.00	

MCI: mild cognitive impairment.

**Table 3:** Mann-Whitney test for intragroup comparisons.

Group	n	Yesavage scale	Median	Percentile 25	Percentile 75	p-value
Healthy/MCI	129	Pre	1.00	1.00	3.00	0.003
	122	Post	2.00	1.00	3.00	0.55

MCI: mild cognitive impairment.

In the social domain, 109 participants (46 healthy and 63 MCI) reported to live with a relative, friend, or acquittance; 44.2% considered that they received good care or attention at home; and 85.3% mentioned not having changes in their relationships with their families as a result of quarantine. In the sample (n=129), around 37.2% responded that they did not have financial resources for household expenses, and 42.6% were concerned about the economic situation of the family.

## DISCUSSION

The primary purpose of this study was to determine the physical, psychological, and social health conditions and mood of COVID-19 quarantine in adults with MCI, who are part of the project “Longitudinal Measurement of the integration function in short-term memory in patients with Mild Cognitive Impairment (MCI).” The results suggested that the quarantine had a negative psychological impact on sleep habits, both in the healthy group and the MCI group. Social distancing and the association of it with loneliness, worries, and low resilience can cause an increase^17^ or decrease in sleep time^18^. People with dementia and insomnia can experience emotional changes (aggressiveness and irritability) as well as symptoms of anxiety and depression^19,20^. In fact, this information corroborated by the results. In the Yesavage scale, the intragroup comparisons of healthy participants were significant (p=0.010) and at the intergroup level the Yesavage scale before and after quarantine showed significant differences in the pretest scores (p=0.003).

The emotional state was mainly affected by quarantine and loneliness. In this study, the participants showed boredom and nervousness as an emotional state, and these are predictors highly related to depression and progression of dementia^20^. However, a tendency to be optimistic was identified, regarding the health crisis caused by the COVID-19 pandemic in more than 50% of the participants in both groups.

Physical health is another condition affected by the restrictions due to the pandemic. About 37% of healthy participants reported not doing physical activities, and they also perceived an increase in symptoms of sickness during quarantine (p=0.043). These findings suggest that physical activity is related to the state of psychological Personal satisfaction^21^. Therefore, involving older people in physical activities could be a protective measure for the older adult population during quarantine^22^.

Social Personal satisfaction is another aspect affected by quarantine, because social interaction is a fundamental factor that helps a person to face an adverse situation. In this research, 109 participants (46 healthy and 63 MCI) reported that they live with the first-degree relatives and stated that they receive good care or assistance from them; results are similar to other research in which it was found that the majority of people live with their family members^17,23,24^ and, to a lesser extent, with a friend or caregiver^25^.

The quarantine due to COVID-19 had a negative psychological effect on both physical and mental health of the participants, mainly affecting the physical domain (physical activity, increased symptoms of physical illnesses), psychological domain (sleep habits, mood, depression), and social domain (tendency to be interested in news related to COVID-19). Statistically significant differences were found at the intergroup and intragroup levels when applying the Yesavage Geriatric Depression Scale before and after quarantine. These results suggest that physical distancing measures and visiting restrictions can reduce the risk of transmission and physical affectations, but it could increase mental distress.

## References

[1] Organización Mundial de la Salud (2020). WHO Director-General’s opening remarks at the media briefing on COVID-19.

[2] Centro Europeo para la Prevención y el Control de Enfermedades (2020). Panel de Situación COVID-19.

[3] Ministerio de salud (2020). “Adultos mayores representan el 49% de las muertes por covid-19 en Colombia”.

[4] Aranda Y, Aranda L, Alcaraz C, Comallonga M (2021). Repercusiones en la salud mental del paciente anciano tras padecer Covid-19: trastorno de estrés postraumático. A propósito de un caso. Rev Esp Geriatr Gerontol.

[5] Cruz Roja Española (2020). Impacto psicológico en las personas mayores por la llegada del SARS-COV2.

[6] Picaza M, Munitis A, Santamaria M, Ozamiz N (2020). Stress, anxiety, and depression in people aged over 60 in the COVID-19 Outbreak in a sample collected in Northern Spain. Am J Geriatr Psychiatry.

[7] Organización Mundial de la Salud (2020). Apoyar a las personas mayores durante la pandemia de COVID-19 es asunto de todos.

[8] Mukhtar S (2020). Psychological impact of COVID-19 on older adults. Curr Med Res Pract.

[9] Santini Z, Jose P, Cornwell E, Koyanagi A, Nielsen L, Hinrichsen C (2020). Social disconnectedness, perceived isolation, and symptoms of depression and anxiety among older Americans (NSHAP): a longitudinal mediation analysis. Lancet Public Health.

[10] Arranz L, Giménez-Llort L, De Castro N, Baeza I, De La Fuente M (2009). El aislamiento social durante la vejez empeora el deterioro cognitivo, conductual e nmunitario. Rev Esp Geriatr Gerontol.

[11] Hartmann-Boyce J, Davies N, Frost R, Park S (2020). Maximizar la movilidad en personas mayores cuando están aisladas con COVID-19.

[12] Huarcaya-Victoria J (2020). Consideraciones sobre la salud mental en la pandemia de COVID-19. Rev Peru Med Exp Salud Publica.

[13] Moreno P, Muñoz C, Pizarro R, Jiménez S (2020). Efectos del ejercicio fisico sobre la calidad del sueño, insomnio y somnolencia diurna en personas mayores. Rev Esp Geriatr Gerontol.

[14] Landis R, Koch G (1977). The measurement of observer agreement for categorical data. Biometrics.

[15] Guerin J, Copersino M, Schretlen D (2018). Clinical utility of the 15-item geriatric depression scale (GDS-15) for use with young and middle-aged adults. J Affect Disord.

[16] Mitchell A, Bird V, Rizzo M, Meader N (2010). Diagnostic validity and added value of the geriatric depression scale for depression in primary care: A meta-analysis of GDS30 and GDS 15. Affect Disord.

[17] Kerstin K (2020). Coping with being cooped up: Social distancing during COVID-19 among 60+ in the United States. Rev Panam Salud Publica.

[18] Grossman ES, Hoffman YSG, Palgi Y, Shrira A (2021). COVID-19 related loneliness and sleep problems in older adults: Worries and resilience as potential moderators. Pers Individ Dif.

[19] Schapira M (2020). Impacto psicosocial de la pandemia por COVID-19 en adultos mayores con demencia y sus cuidadores. Rev Argent Salud Publica.

[20] Robb C, de Jager C, Ahmadi-Abhari S, Giannakopoulou P, Udeh-Momoh C, McKeand J (2020). Associations of Social isolation with anxiety and depression during the early COVID-19 Pandemic: a survey of older adults in London, UK. Front Psychiatry.

[21] Maugeri G, Castrogiovanni P, Battaglia G, Pippi R, D’Agata V, Palma A (2020). The impact of physical activity on psychological health during Covid-19 pandemic in Italy. Heliyon.

[22] Carriedo A, Cecchini J, Fernandez-Rio J, Méndez-Giménez A (2020). COVID-19, psychological well-being and physical activity levels in older adults during the nationwide lockdown in Spain. Am J Geriatr Psychiatry.

[23] Di Santo S, Franchini F, Filiputti B, Martone A, Sannino S (2020). The effects of COVID-19 and quarantine measures on the lifestyles and mental health of people over 60 at increased risk of dementia. Front Psychiatry.

[24] Lee HS, Dean D, Baxter T, Griffith T, Park S (2021). Deterioration of mental health despite successful control of the COVID-19 pandemic in South Korea. Psychiatry Res.

[25] Fernández-Ballesteros R, Sanchez-Izquierdo M (2020). Impacto del COVID-19 en personas mayores en españa: algunos resultados y reflexiones. Clín Salud.

